# Supplementary material for: De-novo protein function prediction using DNA binding and RNA binding proteins as a test case
Source: Nat Commun. 2016 Nov 21;7:13424. doi: 10.1038/ncomms13424 (PMC5121330; doi:10.1038/ncomms13424)
Supplement: Supplementary Information — Supplementary Figure 1-5, Supplementary Table 1-9, and Supplementary References [file ncomms13424-s1.pdf]

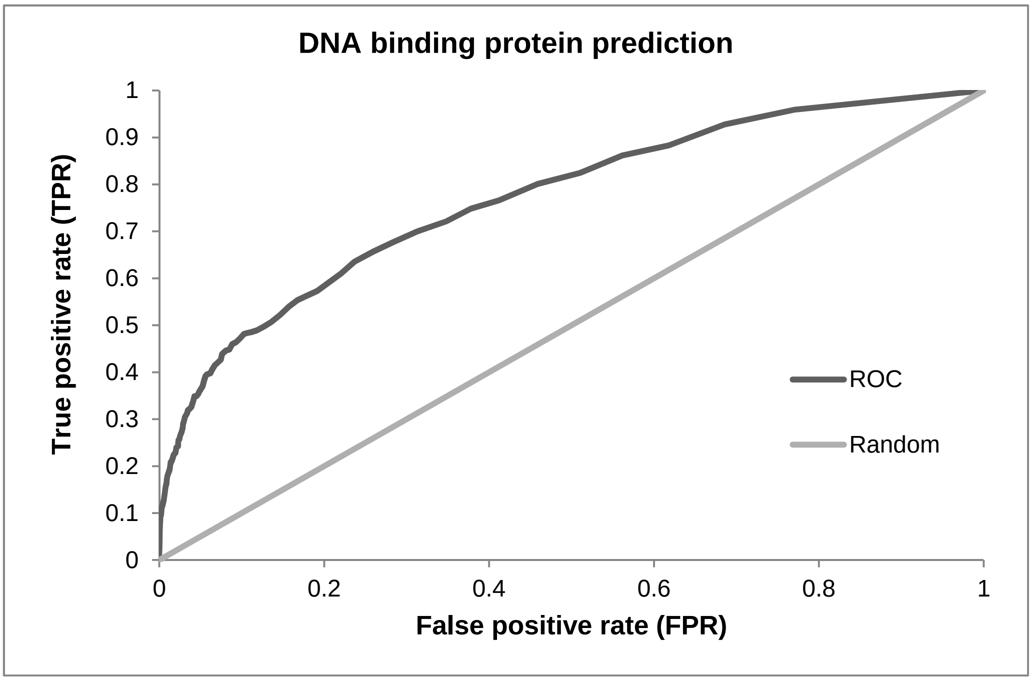

Supplementary Figure 1. Receiver operating characteristic (ROC) curve for predicting whether a protein is DNA-binding or not from DNA-binding residues prediction. Area under curve (AUC) is 0.7699.

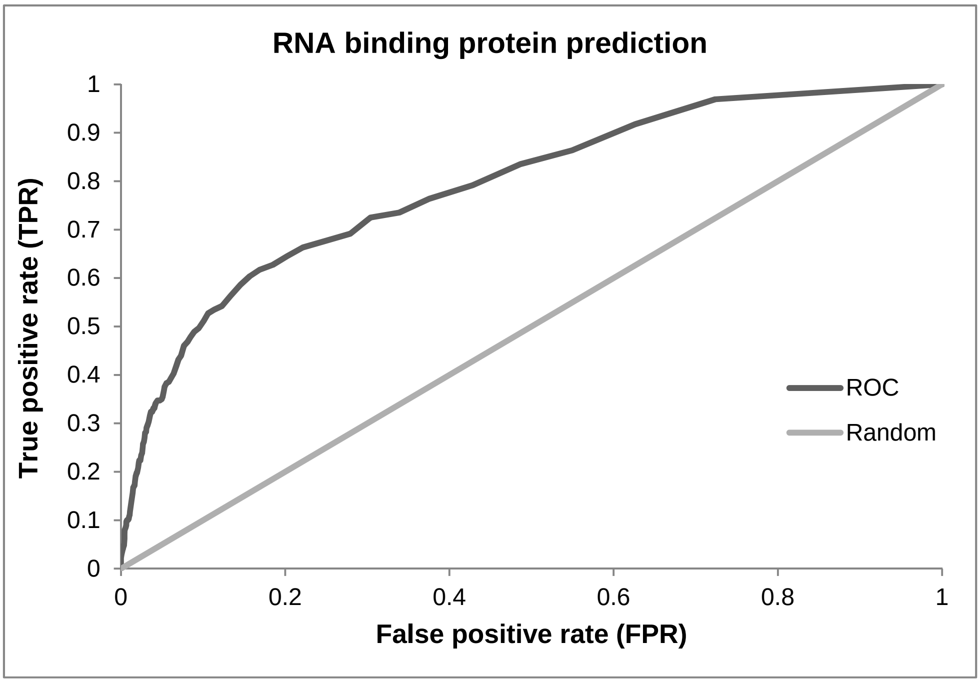

Supplementary Figure 2. Receiver operating characteristic (ROC) curve for predicting whether a protein is RNA-binding or not from RNA-binding residues prediction. Area under curve (AUC) is 0.7898.

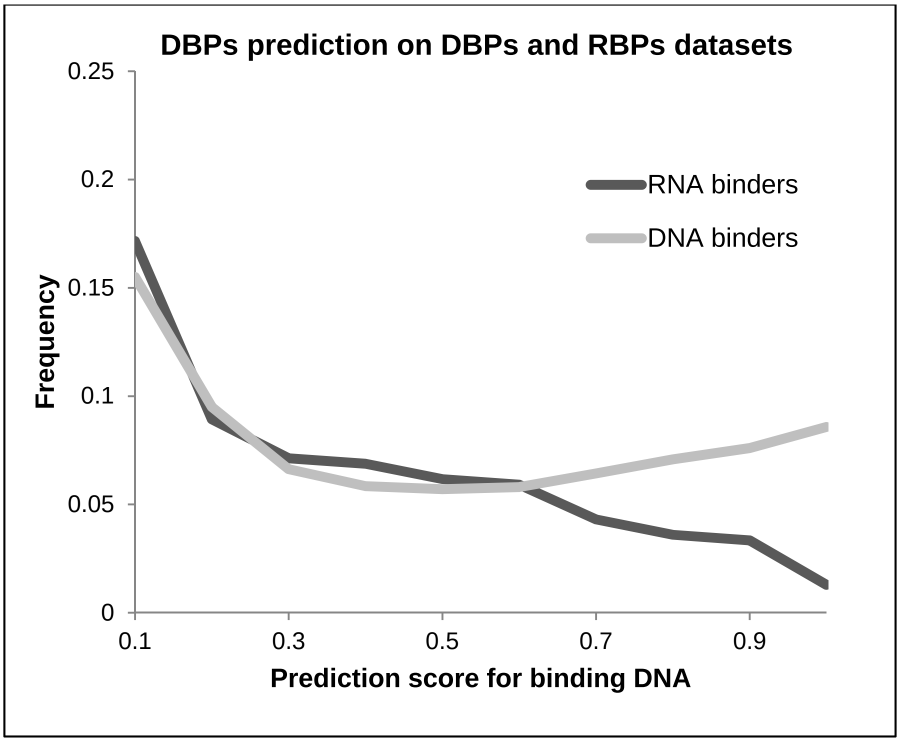

Supplementary Figure 3. Dr. PIP scores for DBPs identification for proteins that are known to bind DNA and for proteins that are known to bind RNA. Prediction scores were binned at intervals of 0.1 and smoothed. DBPs get high scores for binding DNA but very few of the RBPs do.

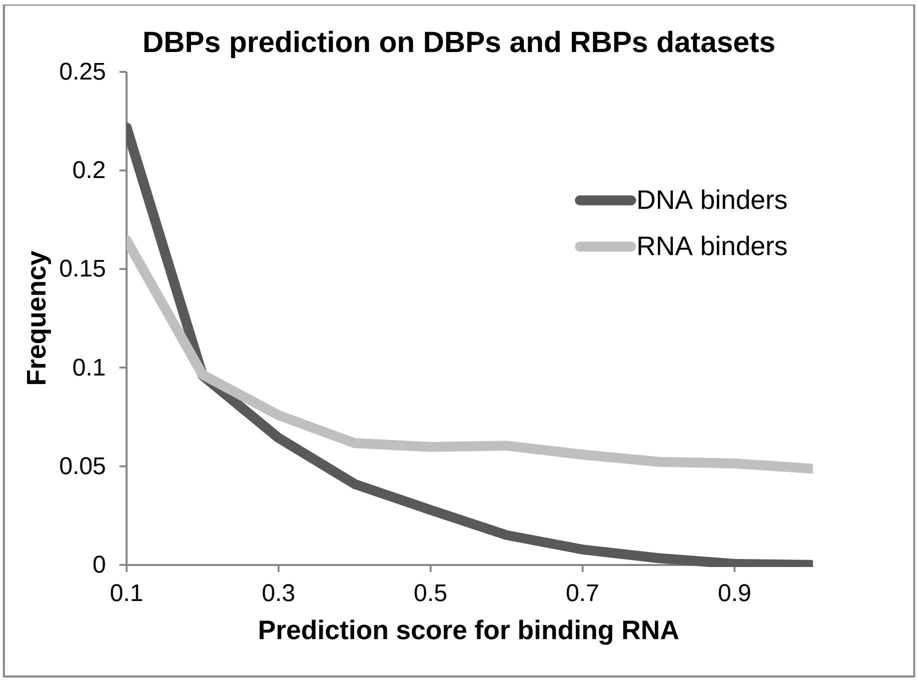

Supplementary Figure 4. Dr. PIP scores for RBPs identification for proteins that are known to bind DNA and for proteins that are known to bind RNA. Prediction scores were binned at intervals of 0.1 and smoothed. RBPs get high scores for binding DNA but almost no DBPs do.

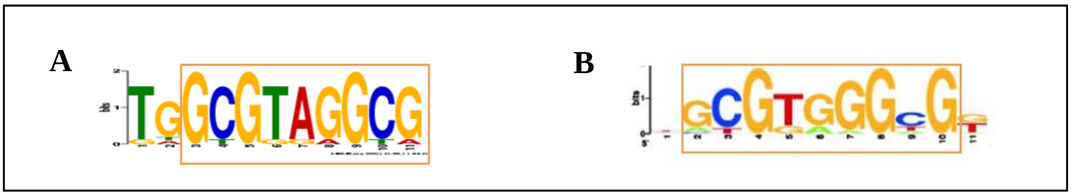

Supplementary Figure 5. Zif268 DNA binding site. **A** After selection, 15 colonies from 4mM 3'AT plate were sequenced and DNA binding motif was generated using MEME. **B** A motif previously generated using B1H[8].

Threshold	Sensitivity	Specificity	FPR	ACC	MCC
0	1	0	1	0.121	0
0.01	0.959	0.229	0.771	0.318	0.152
0.02	0.928	0.314	0.686	0.388	0.175
0.03	0.883	0.382	0.618	0.442	0.181
0.04	0.862	0.438	0.562	0.489	0.199
0.05	0.825	0.49	0.51	0.53	0.206
0.06	0.801	0.541	0.459	0.573	0.223
0.07	0.766	0.588	0.412	0.61	0.232
0.08	0.749	0.622	0.378	0.637	0.244
0.09	0.721	0.652	0.348	0.66	0.249
0.1	0.7	0.687	0.313	0.689	0.263
0.11	0.676	0.717	0.283	0.712	0.273
0.12	0.657	0.741	0.259	0.73	0.281
0.13	0.635	0.763	0.237	0.748	0.288
0.14	0.61	0.78	0.22	0.759	0.287
0.15	0.591	0.795	0.205	0.77	0.289
0.16	0.573	0.809	0.191	0.78	0.292
0.17	0.563	0.82	0.18	0.789	0.299
0.18	0.554	0.832	0.168	0.799	0.307
0.19	0.54	0.843	0.157	0.806	0.31
0.2	0.522	0.853	0.147	0.813	0.311
0.21	0.507	0.864	0.136	0.821	0.314
0.22	0.497	0.873	0.127	0.828	0.32
0.23	0.489	0.882	0.118	0.834	0.327
0.24	0.485	0.888	0.112	0.84	0.335
0.25	0.483	0.894	0.106	0.844	0.342
0.26	0.481	0.897	0.103	0.847	0.347
0.27	0.472	0.902	0.098	0.85	0.348
0.28	0.464	0.907	0.093	0.853	0.35
0.29	0.46	0.912	0.088	0.857	0.356
0.3	0.448	0.915	0.085	0.859	0.354
0.31	0.446	0.919	0.081	0.862	0.36
0.32	0.439	0.924	0.076	0.865	0.364
0.33	0.427	0.925	0.075	0.865	0.357
0.34	0.421	0.929	0.071	0.867	0.359
0.35	0.415	0.933	0.067	0.87	0.363
0.36	0.405	0.936	0.064	0.872	0.363
0.37	0.398	0.938	0.062	0.873	0.361
0.38	0.396	0.942	0.058	0.876	0.369
0.39	0.392	0.944	0.056	0.877	0.371
0.4	0.382	0.946	0.054	0.878	0.366
0.41	0.37	0.948	0.052	0.878	0.36
0.42	0.361	0.951	0.049	0.879	0.36
0.43	0.359	0.952	0.048	0.88	0.361
0.44	0.353	0.953	0.047	0.881	0.36
0.45	0.349	0.955	0.045	0.882	0.362

0.46	0.349	0.957	0.043	0.884	0.369
0.47	0.339	0.959	0.041	0.884	0.364
0.48	0.326	0.961	0.039	0.884	0.358
0.49	0.322	0.963	0.037	0.886	0.361
0.5	0.32	0.965	0.035	0.887	0.366
0.51	0.312	0.967	0.033	0.887	0.363
0.52	0.306	0.968	0.032	0.888	0.363
0.53	0.304	0.969	0.031	0.889	0.363
0.54	0.298	0.97	0.03	0.889	0.36
0.55	0.288	0.971	0.029	0.889	0.356
0.56	0.283	0.972	0.028	0.888	0.351
0.57	0.273	0.973	0.027	0.888	0.346
0.58	0.267	0.974	0.026	0.889	0.345
0.59	0.263	0.975	0.025	0.889	0.344
0.6	0.257	0.976	0.024	0.889	0.34
0.61	0.255	0.977	0.023	0.89	0.343
0.62	0.25	0.977	0.023	0.889	0.339
0.63	0.244	0.977	0.023	0.889	0.332
0.64	0.242	0.978	0.022	0.889	0.331
0.65	0.24	0.979	0.021	0.89	0.337
0.66	0.23	0.98	0.02	0.89	0.33
0.67	0.228	0.98	0.02	0.89	0.329
0.68	0.224	0.983	0.017	0.891	0.334
0.69	0.218	0.984	0.016	0.891	0.333
0.7	0.212	0.984	0.016	0.891	0.33
0.71	0.209	0.986	0.014	0.892	0.331
0.72	0.209	0.986	0.014	0.892	0.336
0.73	0.203	0.987	0.013	0.892	0.33
0.74	0.193	0.987	0.013	0.891	0.323
0.75	0.189	0.988	0.012	0.892	0.323
0.76	0.179	0.99	0.01	0.892	0.322
0.77	0.172	0.991	0.009	0.892	0.317
0.78	0.162	0.991	0.009	0.891	0.308
0.79	0.16	0.992	0.008	0.891	0.309
0.8	0.152	0.993	0.007	0.891	0.304
0.81	0.146	0.993	0.007	0.891	0.298
0.82	0.136	0.994	0.006	0.89	0.29
0.83	0.127	0.995	0.005	0.89	0.282
0.84	0.119	0.996	0.004	0.89	0.28
0.85	0.113	0.997	0.003	0.89	0.281
0.86	0.109	0.997	0.003	0.89	0.281
0.87	0.103	0.997	0.003	0.889	0.271
0.88	0.096	0.998	0.002	0.889	0.264
0.89	0.094	0.999	0.001	0.889	0.271
0.9	0.086	0.999	0.001	0.888	0.258
0.91	0.08	0.999	0.001	0.888	0.251
0.92	0.074	0.999	0.001	0.887	0.244

0.93	0.072	0.999	0.001	0.887	0.241
0.94	0.064	0.999	0.001	0.887	0.23
0.95	0.057	0.999	0.001	0.886	0.214
0.96	0.051	0.999	0.001	0.885	0.202
0.97	0.043	0.999	0.001	0.884	0.184
0.98	0.031	1	0	0.883	0.16
0.99	0.014	1	0	0.881	0.11

Supplementary Table 1. Performance of DNA binding prediction at specific score thresholds.

Threshold	Sensitivity	Specificity	FPR	ACC	MCC
0	1	0	1	0.094	0
0.01	0.969	0.276	0.724	0.341	0.165
0.02	0.918	0.374	0.626	0.425	0.179
0.03	0.864	0.451	0.549	0.49	0.186
0.04	0.835	0.513	0.487	0.544	0.204
0.05	0.792	0.572	0.428	0.592	0.213
0.06	0.763	0.625	0.375	0.638	0.23
0.07	0.735	0.661	0.339	0.668	0.239
0.08	0.725	0.696	0.304	0.699	0.259
0.09	0.692	0.721	0.279	0.718	0.259
0.1	0.676	0.752	0.248	0.745	0.277
0.11	0.663	0.779	0.221	0.768	0.293
0.12	0.645	0.798	0.202	0.783	0.301
0.13	0.627	0.815	0.185	0.798	0.309
0.14	0.617	0.831	0.169	0.811	0.321
0.15	0.604	0.843	0.157	0.82	0.327
0.16	0.586	0.854	0.146	0.829	0.33
0.17	0.563	0.867	0.133	0.838	0.331
0.18	0.542	0.877	0.123	0.846	0.332
0.19	0.535	0.886	0.114	0.853	0.342
0.2	0.527	0.894	0.106	0.859	0.349
0.21	0.512	0.899	0.101	0.863	0.347
0.22	0.496	0.905	0.095	0.867	0.346
0.23	0.488	0.911	0.089	0.871	0.351
0.24	0.478	0.915	0.085	0.874	0.352
0.25	0.468	0.919	0.081	0.877	0.351
0.26	0.46	0.923	0.077	0.88	0.354
0.27	0.44	0.927	0.073	0.881	0.346
0.28	0.432	0.93	0.07	0.883	0.347
0.29	0.419	0.933	0.067	0.884	0.342
0.3	0.404	0.936	0.064	0.886	0.336
0.31	0.393	0.939	0.061	0.888	0.336
0.32	0.386	0.942	0.058	0.889	0.336
0.33	0.383	0.945	0.055	0.892	0.341
0.34	0.375	0.947	0.053	0.893	0.341
0.35	0.368	0.948	0.052	0.893	0.336
0.36	0.355	0.949	0.051	0.893	0.329
0.37	0.35	0.95	0.05	0.894	0.327
0.38	0.347	0.953	0.047	0.896	0.332
0.39	0.347	0.956	0.044	0.898	0.34
0.4	0.342	0.958	0.042	0.9	0.342
0.41	0.337	0.959	0.041	0.9	0.341
0.42	0.332	0.959	0.041	0.9	0.337
0.43	0.332	0.96	0.04	0.901	0.341
0.44	0.326	0.961	0.039	0.902	0.34
0.45	0.324	0.962	0.038	0.902	0.339

0.46	0.324	0.964	0.036	0.903	0.345
0.47	0.316	0.965	0.035	0.903	0.341
0.48	0.303	0.966	0.034	0.904	0.334
0.49	0.296	0.968	0.032	0.904	0.332
0.5	0.29	0.969	0.031	0.905	0.331
0.51	0.283	0.969	0.031	0.905	0.325
0.52	0.28	0.971	0.029	0.906	0.328
0.53	0.27	0.971	0.029	0.905	0.319
0.54	0.262	0.972	0.028	0.905	0.314
0.55	0.257	0.973	0.027	0.906	0.313
0.56	0.252	0.973	0.027	0.905	0.309
0.57	0.239	0.974	0.026	0.905	0.298
0.58	0.234	0.975	0.025	0.905	0.297
0.59	0.224	0.976	0.024	0.905	0.29
0.6	0.224	0.978	0.022	0.907	0.296
0.61	0.219	0.978	0.022	0.907	0.293
0.62	0.208	0.979	0.021	0.906	0.284
0.63	0.198	0.98	0.02	0.907	0.279
0.64	0.198	0.981	0.019	0.907	0.281
0.65	0.19	0.982	0.018	0.908	0.279
0.66	0.18	0.983	0.017	0.907	0.27
0.67	0.172	0.983	0.017	0.907	0.261
0.68	0.17	0.984	0.016	0.908	0.264
0.69	0.167	0.985	0.015	0.908	0.265
0.7	0.157	0.986	0.014	0.908	0.254
0.71	0.147	0.987	0.013	0.907	0.245
0.72	0.141	0.987	0.013	0.907	0.241
0.73	0.139	0.987	0.013	0.907	0.239
0.74	0.134	0.988	0.012	0.907	0.235
0.75	0.123	0.988	0.012	0.907	0.223
0.76	0.123	0.988	0.012	0.907	0.223
0.77	0.118	0.989	0.011	0.907	0.218
0.78	0.111	0.99	0.01	0.907	0.21
0.79	0.108	0.99	0.01	0.907	0.209
0.8	0.103	0.991	0.009	0.907	0.204
0.81	0.1	0.992	0.008	0.908	0.208
0.82	0.1	0.993	0.007	0.908	0.214
0.83	0.098	0.993	0.007	0.909	0.217
0.84	0.09	0.994	0.006	0.908	0.206
0.85	0.085	0.994	0.006	0.909	0.204
0.86	0.08	0.995	0.005	0.909	0.205
0.87	0.072	0.996	0.004	0.909	0.193
0.88	0.062	0.996	0.004	0.908	0.171
0.89	0.049	0.996	0.004	0.907	0.148
0.9	0.049	0.997	0.003	0.907	0.151
0.91	0.046	0.997	0.003	0.907	0.148
0.92	0.041	0.998	0.002	0.907	0.146

0.93	0.041	0.998	0.002	0.908	0.15
0.94	0.036	0.998	0.002	0.908	0.145
0.95	0.031	0.999	0.001	0.908	0.146
0.96	0.028	0.999	0.001	0.908	0.145
0.97	0.023	1	0	0.908	0.145
0.98	0.018	1	0	0.907	0.128
0.99	0.003	1	0	0.906	0.048

Supplementary Table 2. Performance of RNA binding prediction at specific score thresholds.

Gene	Restriction	Forward Primer	Reverse Primer
CH004_HUMAN	KpnI/BamHI	5' TAAGGTACCATGAAAGCAAAGCGAAGCCACCAAG	5' TAAGGATCCTCAGTGAACTTTGATGGAATATTTCCGC
CS043_HUMAN	KpnI/AvrII	5' TAAGGTACCATGGCTGCCCGAGGGAGACGGGCGGAGC	5' TAACCTAGGTCATTTCACCAGGGGCCGAGTTTTATC
CI009_HUMAN	NotI/AvrII	5' TAAGCGGCCGCGGACTACAAGGATGACGACGACAAGTTCCGGACCGGTTCCAAGACACCCCCCCATGGTACCATGAATGAGGTGAAAGAATCCCTTCG	5' TAACCTAGGTTACAATTTCCCACCAGGAGGCCTCC
CC130_HUMAN	NotI/AvrII	5' TAAGCGGCCGCGGACTACAAGGATGACGACGACAAGTTCCGGACCGGTTCCAAGACACCCCCCCATGGTACCATGGGTGAAAGGAAAGGGGTCAACAAGTAC	5' TAACCTAGGTCACTCACTCTCCGAGTCGGAGTAGTCC
IFRD1_HUMAN	KpnI/BamHI	5' TAAGGTACCATGCCGAAGAACAAGAAGCGG	5' TAAGGATCCCTAGAAGAATTCTCCAACATCTGC
GSCR2_HUMAN	KpnI/BamHI	5' TAAGGTACCATGGCGGCAGGAGGCAGTGGCGTTGG	5' TAAGGATCCCTACAACTGGATCTCACGGAACGCCCGC
CN093_HUMAN	KpnI/BamHI	5' TAAGGTACCATGTCCTTCAGTGCCACCATTCTCTTCTC	5' TAAGGATCCTTATTCATCCTTTTCCACCTTG
SOCS4_HUMAN	NotI/AvrII	5' TAAGCGGCCGCGGACTACAAGGATGACGACGACAAGTTCCGGACCGGTTCCAAGACACCCCCCCATGGTACCATGGCAGAAAATAATGAAAATATTAG	5' TAACCTAGGCTAGCATTGCTGTTCTGGTGCATC
MAK16_HUMAN	KpnI/BamHI	5' TAAGGTACCATGCAGTCGGATGATGTTATCTGG	5'TAAGGATCCTCACGTGGTTTTGGCTTTGGCCAC
FGF14_HUMAN	KpnI/BamHI	5' TAAGGTACCATGGCCGCGGCCATCGCTAGC	5' TAAGGATCCTCATGTTGTCTTACTCTTGTTT
FGF18_HUMAN	NotI/AvrII	5' TAAGCGGCCGCGGACTACAAGGATGACGACGACAAGTTCCGGACCGGTTCCAAGACACCCCCCCATGGTACCATGTATTCAGCGCCCTCCGCCTGCAC	5' TAACCTAGGCTAGGCAGGGTGTGTGGGCCGGAT

Supplementary Table 3. Genes whose ORFs were cloned into the pB1H1 vector, their restriction sites and their forward and reverse primers.

Protein	Class	Dr. PIP	DBS-pred[1]	DNAbinder (AA composition)[2]	iDNA-Prot[3]	nDNA-Prot[4]	enDNA-Prot[5]	SVMProt[6]	DNABIND[7]
P0A6X7	Binding	100%	50.1%	1.8409802	+	-	+	+	0.9844
P03069	Binding	91.5%	89.4%	0.8035656	-	-	-	+	0.7334
Q97XK8	Binding	69%	93.4%	-0.1524151	+	-	-	-	0.2449
P03369	Binding	29.8%	42%	0.20011058	-	-	-	+	0.5201
Q8TWU6	Binding	25.9%	4%	0.57886458	-	-	-	+	0.8285
P63000	Binding	25.2%	20.6%	-0.1967644	-	-	-	-	0.2928
Q58718	Binding	22.8%	79.5%	2.4591011	+	-	+	+	0.9563
Q8TDY4	Binding	16.1%	12%	0.40101977	-	-	-	+	0.5136
Q5SLP8	Binding	15.3%	12.1%	2.1406225	+	-	+	-	0.9957
B6ZK72	Non-binding	12.1%	3.9%	-0.5099648	-	-	-	-	0.5792
P58547	Non-binding	12%	15.6%	-4.1796674	-	-	-	-	0.0001
P38713	Non-binding	11.8%	89.5%	1.335912	-	-	+	-	0.8119
P80403	Non-binding	11.4%	95.9%	-2.4237333	-	-	+	-	0.0631
P28881	Non-binding	11.1%	21%	0.90045297	+	-	+	-	0.922
P86794	Non-binding	10.8%	6.6%	3.7889565	+	-	+	-	1
Q8TF42	Non-binding	10.4%	96.5%	0.38700269	-	-	-	-	0.3708
P0CH62	Non-binding	10.2%	12.8%	-1.0663754	-	-	-	-	0.1244
P58606	Non-binding	10%	88.8%	-1.7213793	-	-	-	-	0.2934
P17350	Non-binding	0.3%	66.9%	-0.8814125	-	-	+	-	0.31

Supplementary Table 4. A comparison between DBPs prediction methods with available online web-servers performances for the ORFans DNA-binding and non-DNA binding dataset classification.

1A02_N	1A0A_A	1A1V_A	1A73_A	1AKH_A	1AM9_A	1AN4_A	1AWC_B	1B01_A	1B3T_A	1B72_A	1B72_B	1BDT_A
1BDX_A	1BG1_A	1BHM_A	1BRN_L	1C9B_A	1CBV_L	1CEZ_A	1CF7_A	1CF7_B	1CKT_A	1CMA_A	1CQT_I	1CW0_A
1D02_A	1D2I_A	1D5Y_A	1DC1_A	1DH3_A	1DMU_A	1DNK_A	1DP7_P	1ECR_A	1EO3_A	1EWN_A	1EYU_A	1F0V_A
1F4K_A	1FIU_A	1FOK_A	1FOS_F	1FZP_B	1GD2_E	1GM5_A	1H0M_A	1H88_C	1H9T_A	1HAO_H	1HCR_A	1HJB_A
1HLV_A	1HWT_C	1I3J_A	1I7D_A	1IAW_A	1IXY_A	1J75_A	1JB7_A	1JEY_A	1JEY_B	1JFI_A	1JFI_B	1JT0_A
1K4S_A	1K6O_B	1K78_A	1KBU_A	1KSY_A	1LAU_E	1LBG_A	1LQ1_C	1LWS_A	1M07_A	1MDY_A	1MJE_A	1MM8_A
1MNM_C	1NGM_B	1NKP_A	1NLW_A	1NOP_A	1NVP_D	1ODH_A	1OE4_A	1ORN_A	1OUP_B	1OZJ_A	1P4E_A	1PFI_A
1PP8_F	1PV4_A	1PYI_A	1Q0T_A	1QAI_A	1QBJ_A	1QPI_A	1QRV_A	1QUM_A	1QZG_A	1R71_A	1RB8_F	1RB8_J
1RC8_A	1RH6_B	1RM1_A	1RM1_C	1RRQ_A	1RZ9_A	1SA3_A	1SFU_A	1SKN_P	1T2K_D	1T38_A	1T8E_B	1TAU_A
1TEZ_A	1TF6_A	1TRO_A	1U1K_A	1U3E_M	1U78_A	1U8B_A	1U8R_A	1V14_A	1VAS_A	1VRR_A	1W36_B	1W36_C
1WTE_A	1WVL_A	1X9N_A	1XF2_H	1XHZ_A	1XPX_A	1XSD_A	1Y6F_A	1Z1B_A	1Z63_A	1Z9C_A	1ZME_C	1ZS4_A
1ZZI_A	2A0I_A	2A3V_A	2AJQ_A	2AOQ_A	2AYB_A	2BGW_A	2BNW_A	2BOP_A	2BSQ_A	2BSQ_E	2BZF_A	2C5R_A
2C62_A	2C9L_Y	2CCZ_A	2D5V_A	2DDG_A	2DRP_A	2DWL_A	2ER8_A	2ETW_A	2EX5_A	2EZV_A	2F8N_K	2FIO_A
2FKC_A	2FQZ_A	2G1P_A	2GXA_A	2H27_A	2H7F_X	2H8C_B	2H8R_A	2HZV_A	2I0Q_B	2IBS_A	2IHN_A	2IIE_A
2KN7_A	2KO0_A	2L4L_A	2LEV_A	2LEX_A	2LTT_A	2M2W_A	2MF8_A	2NNY_A	2NRA_C	2O49_A	2O61_A	2O8B_A
2O8B_B	2OAA_A	2ODI_A	2OFI_A	2OST_A	2OWO_A	2OXV_A	2P5L_C	2P6R_A	2PFJ_A	2QFJ_A	2QHB_B	2QL2_B
2QNC_A	2QSH_A	2R0Q_C	2R1J_L	2R8J_A	2R9L_A	2RBA_A	2RBF_A	2V1U_A	2V6E_A	2VE9_A	2VHG_A	2VLA_A
2VTB_A	2VY1_A	2VYE_A	2W35_A	2W42_A	2W7N_A	2WB2_A	2WIW_B	2WT7_A	2X6V_A	2XHI_A	2XRO_A	2XSD_C
2Y35_A	2Y7H_A	2Y7H_B	2Y9Z_A	2Y9Z_B	2YPA_A	2YPA_B	2YVH_A	2Z3X_A	2Z70_A	2Z9O_A	2ZHG_A	3A4K_A
3A5T_A	3A6N_B	3AAF_A	3AN2_A	3AU6_A	3B39_A	3BDN_A	3BEP_A	3BM3_A	3BRF_A	3BS1_A	3C25_A	3C2I_A
3CLC_A	3CMU_A	3COQ_A	3CRO_L	3D2W_A	3D6Y_A	3DLB_A	3DNV_A	3DNV_B	3DPG_A	3DSC_A	3DZU_A	3E0D_A
3E6C_C	3EBC_A	3ERE_D	3EY1_A	3EYI_A	3F2B_A	3FC3_A	3FD2_A	3FDQ_A	3FMT_A	3G73_A	3GFI_A	3GLF_A
3GLF_B	3GNA_A	3GP8_A	3GQC_A	3GX4_X	3GXQ_A	3GZ6_A	3H0D_A	3H15_A	3H25_A	3H8O_A	3HOS_A	3HQF_A
3HQG_A	3HTS_B	3HXO_A	3IAY_A	3IGM_A	3IV5_A	3IYD_A	3IYD_C	3IYD_D	3IYD_F	3JSO_A	3JX7_A	3K4X_A
3K57_A	3K70_D	3KD1_E	3KDE_C	3KHC_B	3KJP_A	3KLH_A	3KNT_A	3LDY_A	3LSP_A	3LWH_A	3M4A_A	3MLP_A
3MVD_K	3MX4_A	3MZH_A	3N1I_A	3N6S_A	3NDH_A	3NGO_A	3NGZ_A	3O9X_A	3OA6_A	3OGD_A	3ON0_A	3OOL_A
3OQG_A	3PIH_A	3POV_A	3PTA_A	3PX7_A	3PZP_A	3Q0A_A	3QE9_Y	3QOQ_A	3QWS_A	3QYX_A	3R7P_A	3R8F_A
3RA4_A	3RMP_A	3RN2_A	3SJM_A	3SLP_A	3SQI_A	3SSC_A	3SWM_A	3SZ5_A	3SZQ_A	3TED_A	3TU4_K	3TWM_A
3U3W_A	3U3Y_A	3U44_A	3U44_B	3U58_A	3U5Z_A	3U5Z_B	3U5Z_F	3U6Y_C	3UBT_Y	3UDG_A	3UGM_A	3UPU_A
3UVF_A	3V1Z_A	3V4R_A	3VAF_A	3VD6_C	3VEA_A	3VH0_A	3VK7_A	3VKE_A	3VOK_A	3VW4_A	3WAZ_A	3WGI_A
3ZH2_A	3ZHM_A	3ZI5_A	3ZKC_A	3ZPL_A	3ZQL_C	3ZVK_E	3ZVM_B	4A12_A	4A15_A	4A3I_A	4A3I_B	4A75_A
4A8Q_A	4AIJ_A	4ASO_A	4ATI_A	4AWL_A	4B1O_Q	4BAC_A	4BHM_A	4BXO_B	4C2T_A	4CGZ_A	4CRO_A	4CSA_C
4D1Q_A	4DAV_A	4DKJ_A	4DQY_C	4E5Z_B	4E9F_A	4EGY_A	4ER8_A	4ESJ_A	4EUW_A	4FCY_A	4FLT_A	4FPV_A
4FTH_A	4FX4_A	4FZX_C	4G0R_A	4G7H_D	4G92_C	4GCK_A	4GDF_A	4GFB_A	4GFH_A	4GLE_A	4GNX_B	4GNX_C
4H10_A	4H5Q_A	4HCB_A	4HF1_A	4HID_A	4HLY_B	4HQB_A	4HQE_A	4HQU_A	4HRI_A	4HT4_A	4I27_A	4I2O_A
4I6Z_A	4IEM_A	4IHS_A	4ITQ_A	4IUF_A	4IX7_A	4J19_A	4J1J_A	4JCX_A	4JL3_A	4KB6_A	4KDP_A	4KFC_A
4KIS_A	4KUD_D	4L0Y_A	4L62_A	4LB5_A	4LD0_A	4LDX_A	4LJR_A	4LLL_A	4LMG_A	4LT5_A	4LUP_A	4LVI_A
4M8B_R	4M8O_A	4MDE_A	4MGU_A	4MTD_A	4MZR_A	4N0O_A	4NDH_A	4NDY_I	4NDY_M	4NI7_A	4NM6_A	4OI7_A
4OMY_A	4OU6_A	4OWW_B	4P0P_A	4P0P_B	4PAR_A	4PE8_A	4POG_A	4PSO_A	4PU3_A	4Q0R_A	4QEN_A	4QPQ_A
4QQW_A	4QTJ_A	4R28_C	4R89_A	4R8P_L	4TUG_A							

Supplementary Table 5. A List of all DNA binding protein chains from the PDB used for the positive dataset of Dr. PIP DBPs prediction.

1A34_A	1A9N_A	1AQ3_A	1AV6_A	1B2M_A	1BMV_2	1CWP_A	1E8O_A	1E8O_B	1EIY_B	1F7U_A	1F8V_E	1FFY_A
1FJG_F	1FJG_H	1GAX_A	1GIY_I	1GIY_X	1H2D_A	1H3E_A	1H4Q_A	1HR0_W	1HVU_A	1I5L_A	1I96_V	1IL2_A
1J2B_A	1J5A_M	1JBR_A	1JID_A	1JJ2_G	1KNZ_A	1L9A_A	1LAJ_A	1N1H_A	1NKW_T	1NKW_Y	1OOA_A	1P6V_A
1P85_R	1Q2R_A	1QF6_A	1RPU_A	1S1H_T	1S1I_A	1S72_2	1S72_L	1SER_A	1U0B_B	1U6P_A	1UN6_C	1UTF_A
1UVI_A	1VFG_A	1VS5_F	1VS5_T	1VS5_U	1VS6_Y	1VSA_V	1VSA_W	1VW4_X	1VW4_Z	1VW9_b	1VW9_c	1VW9_d
1VW9_f	1VW9_g	1VY8_N	1VY8_T	1VY8_U	1VY9_J	1W2B_5	1WMQ_A	1WNE_A	1WZ2_A	1YTU_A	1YVP_A	1YYK_A
1ZBH_A	1ZH5_A	1ZN0_A	2A1R_A	2AAR_7	2ANN_A	2ASB_A	2AZ0_A	2AZX_A	2B3J_A	2B63_A	2B63_B	2BH2_A
2BX2_L	2CSX_A	2D6F_C	2DER_A	2DLC_X	2DU3_A	2F8K_A	2F8S_A	2FK6_A	2FMT_A	2FTC_A	2FTC_F	2FZ2_B
2GIC_A	2GJE_A	2GJE_D	2GJW_A	2GO5_1	2GTT_A	2GXB_A	2HT1_A	2HVY_A	2HVY_C	2I82_A	2IX1_A	2J0Q_T
2J37_W	2JEA_A	2JLU_A	2L3J_A	2LBS_B	2M8D_B	2MF0_A	2MJH_A	2MKK_A	2MKN_A	2MTV_A	2NQP_C	2OZB_B
2PJP_A	2PLY_A	2PY9_B	2Q66_A	2QQP_F	2QUX_A	2R7R_A	2R8S_H	2RD2_A	2RKJ_A	2VNU_D	2W2H_A	2WJ8_K
2WW9_A	2WW9_I	2XFM_A	2XFZ_Y	2XGJ_A	2XLI_A	2XS2_A	2XZM_3	2XZM_4	2XZM_8	2XZM_P	2XZM_R	2XZM_Z
2Y9A_C	2Y9A_D	2Y9A_E	2Y9A_F	2Y9A_G	2YKR_W	2ZI0_A	2ZKO_A	2ZKQ_b	2ZKQ_s	2ZKR_b	2ZKR_c	2ZKR_f
2ZKR_m	2ZKR_o	2ZKR_p	2ZKR_q	2ZKR_t	2ZKR_u	2ZKR_w	2ZM5_A	2ZNI_A	2ZZM_A	3A2K_A	3A6P_A	3A6P_C
3ADI_A	3ADL_A	3AEV_B	3AGV_A	3AHU_A	3AL0_B	3AL0_C	3AM1_A	3AMT_A	3AVT_A	3B0V_C	3BBN_E	3BBN_F
3BBN_T	3BBN_U	3BBO_2	3BBO_3	3BBO_4	3BBO_D	3BBO_P	3BBO_Q	3BBO_T	3BBO_V	3BBO_X	3BBO_Y	3BBO_Z
3BO0_B	3BSN_A	3BT7_A	3CIY_A	3CW1_A	3CW1_L	3D2S_A	3D5B_J	3DD2_H	3DEG_C	3DH3_B	3EPH_A	3EQT_A
3ER9_B	3F1E_X	3G9Y_A	3GPQ_A	3HL2_A	3HTX_A	3HUW_V	3IAB_A	3IAB_B	3ICQ_T	3ID5_A	3IE1_A	3J01_A
3J16_A	3J16_B	3J20_Q	3J20_W	3J36_1	3J44_5	3J44_d	3J44_g	3J44_P	3J5S_D	3J5X_Z	3J5Y_A	3J6V_E
3J6V_F	3J6V_G	3J6V_I	3J6V_J	3J6V_K	3J6V_N	3J6V_O	3J6V_Q	3J6V_R	3J6V_U	3J6V_V	3J7A_L	3J7A_W
3J7A_Y	3J7Y_0	3J7Y_1	3J7Y_3	3J7Y_4	3J7Y_5	3J7Y_6	3J7Y_8	3J7Y_9	3J7Y_a	3J7Y_b	3J7Y_c	3J7Y_d
3J7Y_e	3J7Y_F	3J7Y_g	3J7Y_h	3J7Y_i	3J7Y_I	3J7Y_j	3J7Y_k	3J7Y_L	3J7Y_M	3J7Y_N	3J7Y_o	3J7Y_P
3J7Y_q	3J7Y_Q	3J7Y_R	3J7Y_s	3J7Y_S	3J7Y_T	3J7Y_U	3J7Y_V	3J7Y_X	3J7Y_Z	3J80_i	3J81_j	3J81_k
3J8G_X	3K49_A	3KFU_E	3KFU_J	3KIQ_y	3KS8_A	3KTW_A	3MDG_A	3MOJ_B	3NMU_F	3O58_J	3O58_N	3O8C_A
3OIJ_A	3OUY_A	3PDM_P	3PKM_A	3Q1Q_A	3Q2T_C	3QG9_A	3QJJ_B	3QRP_A	3R2D_A	3RC8_A	3RTJ_A	3RW6_A
3S4G_A	3SN2_A	3SQW_A	3T3O_A	3T5N_A	3TRZ_A	3TUP_A	3U2E_A	3UOQ_W	3UZS_A	3V11_B	3V22_V	3V2C_Y
3VJR_A	3VYX_A	3VYY_A	3W1K_A	3W3S_A	3WBM_A	3WC1_A	3WQY_A	3ZC0_E	3ZGZ_A	4A17_M	4A18_H	4A18_J
4A18_L	4A18_M	4A18_N	4A18_O	4A18_P	4A18_Q	4A18_T	4A18_U	4A18_X	4ADV_V	4ADX_G	4AFY_A	4AM3_A
4ANG_A	4ATO_A	4B3G_A	4B8T_A	4BBL_A	4BHH_B	4BPB_A	4BYB_Y	4BYX_V	4C4W_A	4C9D_A	4CE4_2	4CE4_7
4CE4_I	4CSF_S	4CSU_9	4CXC_E	4DH9_Y	4DWA_A	4E78_A	4EJT_A	4ERD_A	4F1N_A	4F3T_A	4FTE_C	

Supplementary Table 6. A List of all RNA binding protein chains from the PDB used for the positive dataset for Dr. PIP RBPs prediction.

A0MES8	A1Z9E2	A2A884	B6UVW4	C9JV30	F4I443	F4KGY6	O00268	O00571	O00910	O01789	O05797	O08574
O08686	O09106	O14350	O14746	O23116	O24646	O35185	O35473	O35738	O43133	O43889	O44712	O53467
O53478	O53509	O53838	O54788	O54946	O60563	O61016	O70343	O70437	O80931	O81242	O82166	O82307
O88282	O88291	O88509	O88846	O88898	O88939	O88974	O95361	O97159	O97581	P00582	P02833	P06537
P06709	P06766	P06843	P07604	P07664	P07665	P07799	P08044	P08047	P08152	P09775	P09884	P0A3W8
P0A8B5	P0A8P6	P0A8W0	P0A9E5	P0A9F3	P0A9F9	P0A9M0	P0A9U6	P0AAR0	P0ABT2	P0ACG1	P0ACG8	P0ACH5
P0ACH8	P0ACK2	P0ACL5	P0ACM2	P0ACP1	P0ACR9	P0AD01	P0AE39	P0AES4	P0AES6	P0AF10	P0AF20	P0AFI2
P0AGB3	P10505	P10627	P12023	P12980	P13297	P13469	P13902	P14003	P14316	P14375	P14404	P15314
P17208	P17433	P17446	P17671	P18124	P18146	P18591	P18592	P18848	P20023	P20153	P21228	P22260
P22265	P22449	P22523	P22813	P23367	P23441	P23611	P23792	P23862	P24278	P25172	P25208	P25490
P25799	P25932	P25992	P26801	P27245	P28322	P28359	P29374	P29375	P29692	P29747	P30958	P31316
P31380	P32115	P32333	P32591	P32862	P33224	P33228	P33244	P33301	P34021	P35398	P35600	P35631
P35638	P35820	P36417	P37309	P37671	P38532	P38533	P38845	P38935	P39015	P39749	P39964	P40064
P40764	P40791	P40847	P40965	P41073	P41151	P41183	P41772	P41971	P42669	P42736	P42775	P42777
P43079	P43267	P43276	P43351	P43609	P43672	P43680	P45448	P45481	P46668	P47806	P47825	P47988
P48301	P49917	P50105	P50534	P50535	P50750	P51450	P51530	P51843	P52172	P52653	P52655	P53050
P53551	P53564	P53783	P54278	P56671	P56672	P58334	P61216	P61372	P63013	P63201	P63204	P70178
P70279	P70365	P70396	P70403	P70562	P71229	P75811	P75952	P76053	P83949	P86938	P92973	P93015
P96856	P97302	P97303	P97360	P97367	P97431	P97436	P97474	P97481	P98149	P9WF39	P9WF41	P9WF43
P9WGG7	P9WGI1	P9WGJ9	P9WIP7	P9WJ87	P9WMF5	P9WMF9	P9WMG5	P9WMH5	P9WMH7	P9WMI1	P9WMI9	P9WMJ1
P9WMJ5	P9WP49	Q00322	Q00422	Q01593	Q01664	Q02067	Q02248	Q02574	Q02780	Q03172	Q03468	Q04013
Q04206	Q04437	Q04863	Q04887	Q04996	Q05066	Q05738	Q06596	Q06623	Q06629	Q06630	Q07053	Q07231
Q07243	Q0VBL6	Q0VGT2	Q10103	Q10159	Q10328	Q12145	Q12173	Q12510	Q12905	Q13263	Q13422	Q15327
Q15646	Q16236	Q16514	Q16594	Q1PFR7	Q20646	Q24459	Q24478	Q24533	Q24573	Q26263	Q27403	Q32MZ4
Q38847	Q38895	Q38914	Q39008	Q39088	Q39204	Q3UPW2	Q3UV55	Q46866	Q47274	Q4G338	Q505F1	Q54YU6
Q558Z2	Q5A0W9	Q5ABZ2	Q5AFP3	Q5SXI5	Q5T6L1	Q5TM83	Q5W6R4	Q5XEM9	Q60520	Q60644	Q60821	Q60974
Q61079	Q61164	Q61321	Q61324	Q61467	Q61473	Q61624	Q61967	Q62187	Q62233	Q62311	Q62424	Q62431
Q62520	Q63014	Q64249	Q64279	Q64305	Q64317	Q64321	Q66GR6	Q6A028	Q6EVK6	Q6NXH3	Q6P926	Q6PIM9
Q6SJ95	Q6WKZ7	Q6X5Y6	Q6XP49	Q75GR5	Q75NZ0	Q7M3M8	Q7TQ40	Q7XKC5	Q7XYY2	Q7Y0W3	Q80W88	Q80ZI1
Q84J70	Q84K52	Q86YC2	Q8BG99	Q8BGD7	Q8BIF2	Q8BMQ3	Q8BQQ7	Q8BYH0	Q8C092	Q8C5D8	Q8C6P8	Q8CCE9
Q8CCI5	Q8CIV7	Q8GAH9	Q8GY61	Q8GYY1	Q8GZ13	Q8GZM7	Q8H1E4	Q8IN94	Q8INL6	Q8IS98	Q8JZL0	Q8K0H5
Q8K0L9	Q8K1M4	Q8K5C0	Q8L3W1	Q8L500	Q8L9K1	Q8LDC8	Q8LPR5	Q8MSU4	Q8N0W2	Q8N8E2	Q8NFD5	Q8NHW3
Q8NML3	Q8NQ97	Q8R385	Q8R4E9	Q8R4S5	Q8R4U1	Q8R515	Q8RY95	Q8S307	Q8VCG9	Q8VDL9	Q8VDQ7	Q8VI67
Q8VIG1	Q8VIH1	Q8VIP2	Q8VYJ2	Q8W1E3	Q90655	Q91VN6	Q91YE5	Q91ZW1	Q92172	Q921D4	Q921Q8	Q92750
Q92754	Q92794	Q92878	Q93V99	Q93VJ4	Q93WK5	Q946J8	Q95V55	Q96EK4	Q96IT4	Q96JR9	Q99JB0	Q99JX1
Q99LG4	Q99MB7	Q99MR6	Q99ZW2	Q9AR19	Q9BQA5	Q9BRQ8	Q9BYE7	Q9BZ30	Q9C5G0	Q9C5Q2	Q9C882	Q9CA51
Q9D2A5	Q9DBU5	Q9DBY0	Q9DDT5	Q9EPW2	Q9ER74	Q9ERU3	Q9ESU6	Q9FGZ4	Q9FV70	Q9FV71	Q9GZX5	Q9H147
Q9H160	Q9H2V2	Q9H4L7	Q9H712	Q9H786	Q9HCL5	Q9JJR7	Q9JJZ6	Q9JKD9	Q9JMG6	Q9LF82	Q9LKL2	Q9LKZ3
Q9LUK7	Q9LUW6	Q9LW31	Q9LW85	Q9LZS0	Q9M2K4	Q9M4A2	Q9NLA3	Q9NR97	Q9NS56	Q9NS59	Q9NWN9	Q9NYA1
Q9NYF8	Q9NZC4	Q9P2Y4	Q9P7T4	Q9QWV4	Q9QXE7	Q9QXT8	Q9S9P3	Q9SBC5	Q9SEZ1	Q9SGS2	Q9SIC9	Q9SJ56
Q9SKD0	Q9SKX6	Q9SUP6	Q9SX27	Q9SZP1	Q9TYG4	Q9U6H3	Q9UL49	Q9ULG1	Q9UNA2	Q9UUI6	Q9UUM2	Q9UW14
Q9V4A3	Q9VDA6	Q9VDA7	Q9VE51	Q9VLD6	Q9VYG2	Q9W349	Q9W4F2	Q9W4S7	Q9WTY8	Q9WU42	Q9XGN1	Q9Y6K1
Q9Z0R0	Q9Z0X1	Q9Z0Z7	Q9Z1J1	Q9Z1L3	Q9Z1L8	Q9Z248	Q9Z2D8	Q9Z2E2	Q9Z2F6	Q9ZWM9		

Supplementary Table 7. A List of all DNA binding proteins with Gene Ontology DNA-binding association (go:0003677).

Amino acid type	Positive	Negative	Polar	Hydrophobic	H-bonds
DNA binding proteins	0.157	0.128	0.192	0.406	0.524
Protein-DNA interfaces	0.296	0.074	0.252	0.27	0.684
Prediction	0.294	0.078	0.254	0.24	0.675
RNA binding proteins	0.162	0.125	0.181	0.409	0.512
Protein RNA interfaces	0.31	0.08	0.205	0.287	0.647
Prediction	0.311	0.085	0.205	0.265	0.641

Supplementary Table 8. Types of amino acids in protein-NA interfaces and in our predicted binding sites, compared to their propensities in the protein in general.

	Efficiency	3-AT [mM]		
		**2**	**3**	**4**
pB1H1 empty + pH3U3 empty	$1.4*10^6$	0	0	0
pB1H1 empty + ph3u3 lib	$4*10^7$	0	0	0
pB1H1 Zif268 + pH3U3 empty	$2.6*10^7$	2	0	0
pB1H1 Zif268 + ph3u3 lib	$2.6*10^7$	>1800	>1000	230

Supplementary Table 9. Results of the validation selection: Number of surviving colonies on selective 3-AT plates using B1H assay. Survival represents DNA binding. Zif268 pB1H1 containing cells were transformed with either empty or library containing pH3u3 plasmid and cells were grown on 3-AT selection plates. For negative control, empty pB1H1 was used.

Supplementary references

1. Ahmad, S., Gromiha, M.M. & Sarai, A. Analysis and prediction of DNA-binding proteins and their binding residues based on composition, sequence and structural information. *Bioinformatics* **20**, 477-486 (2004).
2. Kumar, M., Gromiha, M.M. & Raghava, G.P. Identification of DNA-binding proteins using support vector machines and evolutionary profiles. *BMC Bioinformatics* **8**, 463 (2007).
3. Lin, W.Z., Fang, J.A., Xiao, X. & Chou, K.C. iDNA-Prot: identification of DNA binding proteins using random forest with grey model. *PLoS One* **6**, e24756 (2011).
4. Song, L. et al. nDNA-Prot: identification of DNA-binding proteins based on unbalanced classification. *BMC Bioinformatics* **15**, 298 (2014).
5. Xu, R. et al. enDNA-Prot: identification of DNA-binding proteins by applying ensemble learning. *Biomed Res Int* **2014**, 294279 (2014).
6. Cai, C.Z., Han, L.Y., Ji, Z.L., Chen, X. & Chen, Y.Z. SVM-Prot: Web-based support vector machine software for functional classification of a protein from its primary sequence. *Nucleic Acids Res* **31**, 3692-3697 (2003).
7. Szilágyi, A. & Skolnick, J. Efficient prediction of nucleic acid binding function from low-resolution protein structures. *J Mol Biol* **358**, 922-933 (2006).
8. Meng, X., Brodsky, M.H. & Wolfe, S.A. A bacterial one-hybrid system for determining the DNA-binding specificity of transcription factors. *Nat Biotechnol* **23**, 988-994 (2005).
